# Combining PSA density and imaging data with transcript markers from urinary cells to improve prediction of prostate cancer reclassification in patients on active surveillance

**DOI:** 10.1002/bco2.70276

**Published:** 2026-08-20

**Authors:** Angelika Borkowetz, Sebastian Gräfe, Jeremy Kwe, Susanne Fuessel, Christian Thomas, Kati Erdmann

**Affiliations:** ^1^ Department of Urology, Faculty of Medicine and University Hospital Carl Gustav Carus Technische Universität Dresden Dresden Germany; ^2^ German Cancer Consortium (DKTK) Dresden Germany; ^3^ Department of Urology University Hospital Rostock, University Rostock Rostock Germany; ^4^ National Center for Tumor Diseases (NCT) Dresden Germany

**Keywords:** active surveillance, liquid biopsy, non‐invasive biomarkers, prostate cancer, risk reclassification, urinary biomarkers

## Abstract

**Objectives:**

To investigate selected transcripts from urinary cells regarding their potential to predict risk reclassification in patients with prostate cancer (PCa) on active surveillance (AS).

**Patients and methods:**

Urine samples were prospectively collected from 74 patients before control biopsy and immediately processed to obtain urinary cells. A panel of 29 PCa‐associated transcripts with PPIA, RPLP0 and TBP as reference genes was determined by quantitative PCR following multiplexed reverse transcription of RNA with a pre‐amplification step. Receiver operating characteristic (ROC) curve analyses including the calculation of the area under the curve (AUC) were conducted to assess the predictive ability for reclassification of the respective parameters. A multivariate regression analysis was performed to identify independent predictors suitable for combination.

**Results:**

Overall, 31 patients (42%) showed PCa risk reclassification (defined by ISUP group 1 with PSA > 10 ng/mL or ISUP group 2–5 with any PSA level) at control biopsy. The AUC values and accuracies of PSA, PSA density, PSA velocity and maxPI‐RADS for indicating PCa risk reclassification ranged between 0.738 and 0.767 and 66% and 73%, respectively. Of the investigated transcripts, a predictive potential could be shown for MALAT1, MCL1, NEAT1, STAT3 and STAT5B (AUC = 0.622–0.713, accuracies 64%–73%). A model combining PSA density, maxPI‐RADS and the transcript MCL1 resulted in an AUC of 0.828 with a sensitivity, specificity and accuracy of 58%, 93% and 78%, respectively.

**Conclusion:**

These findings highlight the potential of urinary transcripts, particularly in combinations with clinical parameters, for the non‐invasive monitoring of PCa during AS.

## 1 INTRODUCTION

Low‐ and early intermediate‐risk prostate cancers (PCa) are primarily managed by active surveillance (AS) to avoid therapy‐associated complications of definitive treatment while upholding men's quality of life. Selection criteria for AS are mainly based on the serum level of the prostate‐specific antigen (PSA), PSA density (PSAD; serum PSA/prostate volume) and the local tumour staging as well as the initial tumour grading (International Society of Urological Pathology [ISUP]) and number of positive biopsy cores.^1^, ^2^ The timely detection of tumour progression and risk reclassification is crucial and accomplished by regular monitoring of AS patients, which includes frequent PSA measurements, magnetic resonance imaging (MRI) of the prostate and repeat biopsies.^1^ However, repeated biopsies are associated with various complications, such as infections or bleeding, which could lead to non‐adherence to the AS protocol.^3^, ^4^ Furthermore, the multifocality and heterogeneity of PCa can cause a bias in tissue biopsy sampling.^5^ Consequently, AS holds the risk of delaying definitive treatment and thus curability, particularly when patients were misclassified at AS enrollment.

In addition to achieving precise PCa diagnosis before AS enrollment, the identification of clinical or molecular biomarkers is crucial to reliably predict PCa reclassification as early as possible to better stratify patients during AS. PSA derivatives, such as PSAD and PSA kinetics, exhibited an improved predictiveness of ISUP reclassification during AS compared to PSA.^6^, ^7^ Elevated values for PSAD or PSA kinetics were associated with higher reclassification rates.^6^, ^7^, ^8^ Furthermore, patients with tumour‐suspicious MRI lesions of higher degree also demonstrated an increased probability of PCa reclassification.^8^, ^9^ Although risk stratification is further improved by combining PSAD and MRI results,^8^, ^9^, ^10^, ^11^ the risk of reclassification is not negligible in case of low PSAD and negative MRI.^10^

Therefore, the predictive value of clinical and imaging parameters could be complemented by molecular biomarkers, for example, from liquid biopsies.^12^, ^13^ Notably, the analysis of non‐invasively obtained urinary components provides a more specific assessment of PCa due to the anatomic proximity of the urinary system to the prostate.^5^, ^14^ Particularly, PCa‐derived cells or extracellular vesicles (EVs) from urine reflect the molecular pattern of their cancerous origin and thus represent an attractive source for biomarker studies.^14^ In our previous work, we investigated a panel of 29 transcripts from urinary EVs regarding their potential to predict risk reclassification in PCa patients on AS.^15^ The transcript panel consisted of mRNAs and long non‐coding RNAs (lncRNAs) with known differential expression in PCa and/or functional involvement in important biological processes (e.g., apoptosis, cell cycle, transcriptional regulation). Following combination of PSAD, MRI data and transcript markers (AMACR, MALAT1, PCAT29), the predictive power of the individual markers could be surpassed.^15^

In contrast to urinary EVs, urinary cells are easier to collect and process rendering them also a useful source for biomarker analysis. Therefore, we evaluated the predictive potential of the PCa‐related transcript panel from our previous study^15^ in urinary cells from 74 PCa patients on AS monitored by control biopsy. Furthermore, we investigated if the combination of individual urinary biomarkers with clinical and imaging parameters could enhance the predictive power to indicate PCa risk reclassification.

## 2 PATIENTS AND METHODS

### 2.1 Patient cohort

The study included 74 patients with low‐ or early intermediate‐risk PCa managed by AS and scheduled for control biopsy. Control biopsies were defined as confirmatory biopsy as well as any further biopsy during the AS follow‐up. In most patients, the confirmatory biopsy was equal to first control biopsy following AS initiation. Prospective collection and analysis of urine were approved by the institutional review board of the Technische Universität Dresden (EK346082016 and EK123032019) and conducted according to the Declaration of Helsinki. Written informed consent was obtained from all patients.

Suspicious MRI lesions were evaluated according to the Prostate Imaging Reporting and Data System (PI‐RADS) v2.1 with determination of the maximal PI‐RADS score (maxPI‐RADS). A 12‐core systematic biopsy was performed on all patients, while patients with maxPI‐RADS ≥3 lesions received an additional targeted MRI/ultrasound fusion biopsy. Patients with tumour‐free biopsies or detection of ISUP 1 with PSA ≤ 10 ng/mL comprised the stable disease group. Risk reclassification of PCa was defined as an ISUP upgrading to ISUP ≥2 and/or a PSA increase >10 ng/mL in case of stable ISUP 1. Patient characteristics including ISUP grading, PSA, PSAD, PSA velocity (PSAV; defined as PSA change over time from 1st PCa diagnosis to control biopsy) and MRI maxPI‐RADS were documented in a REDCap (Research Electronic Data Capture) data base.^16^, ^17^

### 2.2 Collection and processing of urine samples

Urine was collected following digital‐rectal examination (DRE) at maximum 29 days before control biopsy. Urine samples were processed immediately as described previously^18^ to retrieve the cellular pellet, which was resuspended in 1400 μL QIAzol Lysis Reagent (Qiagen, Hilden, Germany), aliquoted and stored at −80°C until RNA isolation.

### 2.3 RNA isolation, cDNA synthesis, pre‐amplification and quantitative polymerase chain reaction (qPCR)

Total RNA was extracted from one aliquot (700 μL) using the Direct‐zol RNA MiniPrep Kit (Zymo Research, Freiburg, Germany) according to the manufacturer's recommendations. RNA was quantified with the NanoDrop 2000c spectrophotometer (PEQLAB, Erlangen, Germany).

Synthesis of cDNA with subsequent pre‐amplification was conducted as described previously.^15^ Urinary transcripts from pre‐amplified cDNA (1:20 diluted) were then quantified by qPCR using specific TaqMan Gene Expression Assays (Thermo Fisher Scientific, Darmstadt, Germany). All assay IDs and experimental conditions were described in Erdmann et al.^15^ Per sample, averaged crossing point (CP) values from two independent qPCR runs were used to compute relative transcript expression by the ΔΔCP method with normalization to the geometric CP mean of the reference genes PPIA, RPLP0 and TBP (geoM).

### 2.4 Statistical analysis

GraphPad Prism 10.2.3 (GraphPad Software, San Diego, CA, USA) and IBM SPSS Statistics 29.0.0.0 (IBM, Armonk, NY, USA) were used for statistical analysis. Two groups were compared by using the Mann–Whitney *U* test (continuous variables) or Fisher's exact test (categorized variables). Receiver operating characteristic (ROC) curve analysis was used to calculate the area under the curve (AUC) values with their 95% confidence intervals (95% CI) in order to assess the predictive potential of individual or combined parameters. The sensitivity (SNS), specificity (SPC), positive predictive value (PPV), negative predictive value (NPV) and accuracy (ACC) for risk reclassification were calculated by using either established or optimal cut‐off values according to the Youden index. Odds ratios (OR) were determined by univariate logistic regression for all parameters with *p* < 0.1 in ROC curve analysis followed by a multivariate regression in a forward stepwise fashion to identify independent predictors suitable for combination. The predictive value of marker combinations was then evaluated via ROC curve analysis based on sum scores as described previously.^15^ A statistical significance was considered for *p* < 0.05.

## 3 RESULTS

### 3.1 Characteristics of patient cohort and predictive potential of clinical parameters

**FIGURE 1:**
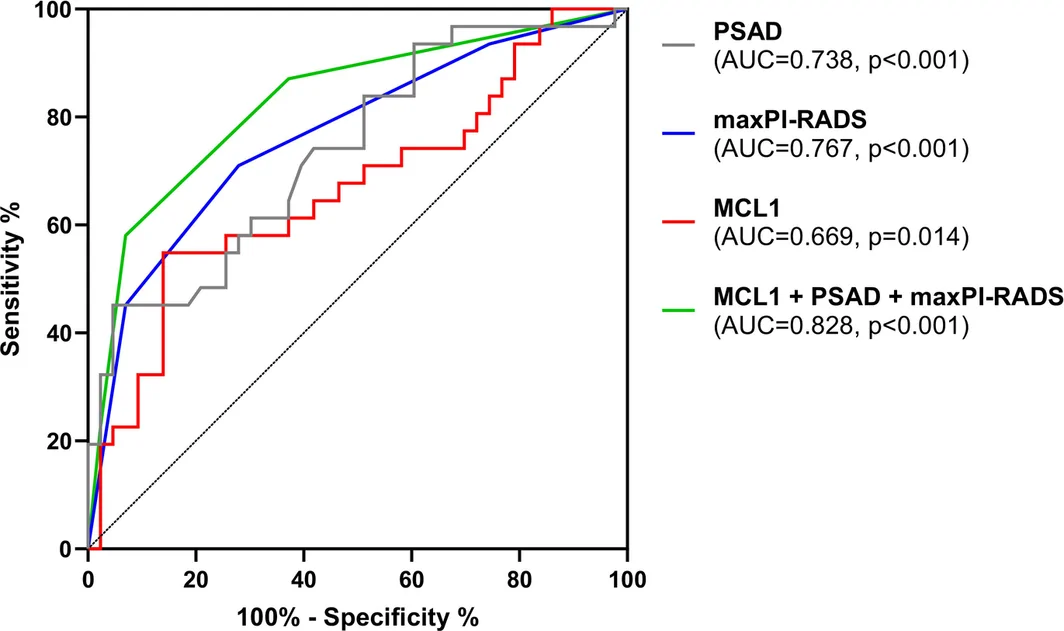
ROC curve analysis of PSAD, maxPI‐RADS and MCL1 alone as well as of their combination to discriminate between stable disease and PCa risk reclassification. AUC, area under the curve; maxPI‐RADS, maximal Prostate Imaging Reporting and Data System score; MCL1, MCL1 apoptosis regulator; PCa, prostate cancer; PSAD, prostate‐specific antigen density; ROC, receiver operating characteristic.

Patients (*n* = 74) were first diagnosed with low‐ or early intermediate‐risk PCa either by scheduled biopsy or incidentally by transurethral resection of prostate tissue (TUR‐P) due to benign prostate hyperplasia (Tables 1 and S1). All patients with ISUP 2 PCa were diagnosed by TUR‐P. Subsequently, all patients were managed by AS, and 31 patients (42%) later showed PCa risk reclassification at control biopsy (Table 1). The time from first PCa diagnosis to the present control biopsy was slightly longer in the reclassified group than in the stable group (1.7 vs. 1.4 years, *p* = 0.025). However, 47 (64%) patients received the present control biopsy within 2 years following initial PCa diagnosis. The number of prior control biopsies on AS did not differ between both groups, and the majority of patients (*n* = 50, 68%) received their first control biopsy during AS follow‐up at the time of study inclusion. In addition to significantly higher PSA, PSAD and PSAV values, patients with PCa risk reclassification showed a higher percentage of tumour‐suspicious lesions in MRI (maxPI‐RADS ≥4) before control biopsy than patients with stable disease (71% vs. 28%; *p* < 0.001; Table 1). Consequently, these clinical parameters could predict PCa risk reclassification with AUC values of 0.738–0.767 (*p* < 0.05) (Table 2 and Figures 1 and S1). By using established cut‐off values (PSA > 10 ng/mL, PSAD >0.2 ng/mL^2^, PSAV >0 ng/mL/year, maxPI‐RADS ≥4), the respective ACC values ranged from 66% to 73% (Table 2 and Figure S1).

**TABLE 1:** Description of the study cohort.

Parameter	Stable *n* = 43	Reclassified *n* = 31	*p* value^a^
**(1) Status at 1st PCa diagnosis and initiation of AS**			
**Diagnosis of PCa**			NA
Biopsy	28	28	
TUR‐P	15	3	
**Median age** (years)	62	68	**0.007**
(Range)	(51–80)	(52–78)	
**Median PSA** (ng/mL)	5.69	6.33	0.111
(Range)	(0.92–13.2)	(2.36–12.80)	
**ISUP grading group**			NA
1 (GS ≤ 6)	39	30	
2 (GS = 3 + 4)	4	1	
**(2) Status at control biopsy on AS**			
**Median time from PCa diagnosis** (years)	1.4	1.7	**0.025**
(Range)	(0.3–10.3)	(0.5–12.1)	
**Time from PCa diagnosis**			0.089
≤2 years	31	16	
>2 years	12	15	
**Number of prior control biopsies**	2 unknown	6 unknown	>0.999
0	31	19	
1–3	10	6	
**Median age** (years)	64	72	**<0.001**
(Range)	(55–81)	(53–84)	
**Median PSA** (ng/mL)	4.82	8.18	**<0.001**
(Range)	(0.36–13.5)	(0.49–24.8)	
**PSA**			**0.003**
≤10 ng/mL	40	20	
>10 ng/mL	3^b^	11	
**Median PSAD** (ng/mL^2^)	0.107	0.147	**<0.001**
(Range)	(0.002–0.313)	(0.019–0.560)	
**PSAD**			**<0.001**
≤0.2 ng/mL^2^	41	18	
>0.2 ng/mL^2^	2	13	
**Median PSAV** (ng/mL/year)	−0.18	0.86	**<0.001**
(Range)	(−12.18–3.66)	(−3.52–5.41)	
**PSAV**			**0.001**
≤0 ng/mL/year	23	5	
>0 ng/mL/year	20	26	
**DRE (cT)**	1 unknown		0.481
Non‐suspicious (cT1)	38	26	
Suspicious (cT2–4)	4	5	
**Median MRI Lesions**	1	1	**0.007**
(Range)	(0–3)	(0–3)	
**MRI maxPI‐RADS**			**<0.001** (2 + 3 vs. 4 + 5)
2	11	2	
3	20	7	
4	9	8	
5	3	14	
**Control biopsy**			0.060
Systematic	11	2	
Systematic and targeted	32	29	
**Median biopsy cores**	16	16	0.245
(Range)	(10–18)	(12–20)	
**Median positive biopsy cores**	0	3	**<0.001**
(Range)	(0–3)	(1–15)	
**ISUP grading group**			NA
Tumour‐free	26		
1 (GS ≤ 6) and PSA ≤ 10 ng/mL	17		
1 (GS ≤ 6) and PSA > 10 ng/mL		4	
2 (GS = 3 + 4)		22	
3 (GS = 4 + 3)		1	
4 (GS = 8)		1	
5 (GS ≥ 9)		3	

Abbreviations: AS, active surveillance; cT, clinical tumour stage; DRE, digital‐rectal examination; GS, Gleason Score; ISUP, International Society of Urological Pathology; maxPI‐RADS, maximal Prostate Imaging Reporting and Data System score; MRI, magnetic resonance imaging; NA, not applicable; PSA, prostate‐specific antigen; PSAD, prostate‐specific antigen density; PSAV, prostate‐specific antigen velocity.

^a^ *P* values were calculated by the Mann–Whitney *U* test (continuous variables) or Fisher's exact test (categorized variables) and highlighted in bold to indicate statistical significance.

^b^ Patients with tumour‐free control biopsy.

**TABLE 2:** Predictive potential of PSAD, maxPI‐RADS and MCL1 alone and in combination to discriminate between stable disease and PCa risk reclassification.

Parameter	PSAD	maxPI‐RADS	MCL1	MCL1 + PSAD + maxPI‐RADS
AUC	0.738	0.767	0.669	0.828
(95% CI)	(0.623–0.853)	(0.655–0.879)	(0.541–0.798)	(0.729–0.927)
*p* value^a^	**<0.001**	**<0.001**	**0.014**	**<0.001**
Cut‐off^b^	>0.2 ng/mL^2^	≥4	≤3.78	2 of 3^c^
SNS (%)	41.9	71.0	54.8	58.1
SPC (%)	95.3	72.1	86.0	93.0
PPV (%)	86.7	64.7	73.9	85.7
NPV (%)	69.5	77.5	72.5	75.5
ACC (%)	73.0	71.6	73.0	78.4

Abbreviations: ACC, accuracy; AUC, area under the curve; CI, confidence interval; maxPI‐RADS, maximal Prostate Imaging Reporting and Data System score; MCL1, MCL1 apoptosis regulator; NPV, negative predictive value; PCa, prostate cancer; PPV, positive predictive value; PSAD, prostate‐specific antigen density; ROC, receiver operating characteristic; SNS, sensitivity; SPC, specificity.

^a^ *P* values were calculated by ROC curve analysis and highlighted in bold to indicate statistical significance.

^b^ For PSAD, the established cut‐off value was used,^2^ while optimal cut‐off values were determined based on the Youden index for the other parameters.

^c^ At least two of three risk factors must be present to indicate PCa risk reclassification.

### 3.2 Deregulation and predictive potential of urinary transcripts

The transcripts MCL1, NEAT1 and STAT3 were significantly (*p* < 0.05) down‐regulated in urinary cells from reclassified patients compared to patients with stable disease, whereas MALAT1 and STAT5B showed evidence of down‐regulation without meeting conventional levels of statistical significance (0.05 ≥ *p* < 0.1; Table S2). The strongest down‐regulation by about 1.7‐fold was observed for MCL1 and STAT3. In addition, these five transcripts could predict PCa risk reclassification with AUC and ACC values ranging between 0.622 and 0.713 and 64% and 73%, respectively (Figures 1 and S1 and Table 2). All other transcripts showed no differential expression and thus were without predictive potential for PCa risk reclassification (Tables S2 and S3).

### 3.3 Increased predictive potential of combined clinical and transcript markers

After confirming the predictive potential of the above‐mentioned clinical and transcript markers by univariate logistic regression (OR = 3.1–14.8, *p* < 0.05), PSAD, maxPI‐RADS and MCL1 were identified as independent predictors for PCa risk reclassification via multivariate logistic regression (Table 3). To achieve an enhanced predictive power, these three markers were combined by generating sum scores per patient according to whether each of the included markers predicted reclassification (1) or not (0). Compared to the individual markers, the combination of MCL1, PSAD and maxPI‐RADS led to a higher AUC value of 0.828 (*p* < 0.001) and ACC of 78% (Table 2 and Figure 1). The improved discriminatory power was reflected by moderate to high values of SNS, SPC, PPV and NPV (Table 2).

**TABLE 3:** Binary logistic regression analysis of selected clinical parameters and urinary transcripts to predict PCa risk reclassification.

Parameter	Univariate analysis			Multivariate analysis^a^		
	OR	95% CI	*p* value^b^	OR	95% CI	*p* value^b^
PSA (reference ≤10 ng/mL)	7.33	1.84–29.29	**0.005**			
PSAD (reference ≤0.2 ng/mL^2^)	14.81	3.02–72.49	**0.001**	10.10	1.79–57.00	**0.009**
PSAV (reference ≤0 ng/mL/year)	5.98	1.93–18.50	**0.002**			
maxPI‐RADS (reference ≤3)	6.32	2.27–17.56	**<0.001**	3.79	1.17–12.21	**0.026**
MALAT1 (reference ≥111.0)	3.07	1.17–8.02	**0.022**			
MCL1 (reference ≥3.78)	7.49	2.45–22.85	**<0.001**	4.14	1.16–14.87	**0.029**
NEAT1 (reference ≥25.70)	5.49	1.70–17.76	**0.004**			
STAT3 (reference ≥0.82)	4.59	1.65–12.72	**0.003**			
STAT5B (reference ≥0.10)	3.71	1.26–10.93	**0.017**			

Abbreviations: CI, confidence interval; MALAT1, metastasis associated lung adenocarcinoma transcript 1; maxPI‐RADS, maximal Prostate Imaging Reporting and Data System score; MCL1, MCL1 apoptosis regulator; NEAT1, nuclear paraspeckle assembly transcript 1; OR, odds ratio; PCa, prostate cancer; PSA, prostate‐specific antigen; PSAD, prostate‐specific antigen density; PSAV, prostate‐specific antigen velocity; STAT3, signal transducer and activator of transcription 3; STAT5B, signal transducer and activator of transcription 5B.

^a^ Multivariate analysis was performed in a forward stepwise fashion using all parameters listed for univariate analysis.

^b^ *P* values indicating a statistical significance are depicted in bold.

## 4 DISCUSSION

Incorporating clinical and imaging parameters, such as PSAD, PSA kinetics and MRI data, could better stratify patients before AS control biopsy according to the risk of reclassification. However, none of these parameters alone can reliably predict PCa reclassification.^10^, ^19^ Combinations with molecular biomarkers from liquid biopsies, such as urine and blood, could improve the predictability of PCa reclassification and thus allow a more personalized AS approach. Therefore, we evaluated the predictive potential of a previously established panel of 29 PCa‐related transcripts^15^ in urinary cells from 74 PCa patients on AS monitored by control biopsy. Of the investigated transcripts, a predictive potential could be shown for MALAT1, MCL1, NEAT1, STAT3 and STAT5B (AUC = 0.622–0.713, ACC = 64%–73%). Apart from MALAT1, these predictive biomarker candidates differed from our previous results in urine EVs from patients on AS, where AMACR, HPN, MALAT1, PCA3 and PCAT29 were identified (AUC = 0.614–0.655, ACC = 61%–68%).^15^ Furthermore, the candidates from urinary cells showed a slightly higher predictive potential than those from urinary EVs. In contrast, the predictive potential of the clinical characteristics PSA, PSAD, PSAV and maxPI‐RADS (AUC = 0.738–0.767, ACC = 66%–73%) was similar to the previous study.^15^ In accordance, various studies have also shown that MRI lesions of higher degree as well as increased PSAD or PSA kinetics were associated with higher reclassification rates.^6^, ^7^, ^8^, ^9^

Following multivariate regression analysis, a model combining PSAD, maxPI‐RADS and the transcript MCL1 surpassed the predictive power of the individual parameters (AUC = 0.828, ACC = 78%). Previously, the combination of PSAD and maxPI‐RADS with the transcripts AMACR, MALAT1 and PCAT1 from urinary EVs also resulted in a highly improved predictiveness (AUC = 0.867, ACC = 85%),^15^ which was more pronounced than in the present study. Notably, PSAD and maxPI‐RADS emerged as clinical parameters with the best predictive power in both studies. Similarly, various studies have shown that the combination of PSAD with MRI data may improve their predictive accuracy for reclassification.^8^, ^9^, ^10^, ^11^ However, the risk of reclassification is not negligible even in patients with low PSAD and negative MRI (maxPI‐RADS <3).^10^

The transcript *MCL1* (MCL1 apoptosis regulator) is a member of the BCL2 family and known to be deregulated in PCa.^20^, ^21^ Its tissue expression was identified as an independent prognostic factor for biochemical recurrence in patients with clinically localized PCa after radical prostatectomy.^21^ However, the expression of *MCL1* has not been evaluated in urinary cells from PCa patients so far, let alone in the setting of AS monitoring. In contrast to clinical and imaging parameters, studies about urinary transcript biomarkers for AS monitoring are scarce. The SelectMDx test (MDxHealth Inc., Irvine, CA, USA), which analyses the mRNA levels of *DLX1* (distal‐less homeobox 1) and *HOXC6* (homeobox C6) in urinary cells to detect high‐risk PCa, has also been evaluated in the context of AS monitoring.^22^ The study by Pepe et al. demonstrated that MRI outperformed the predictive accuracy of the SelectMDx test (85% vs. 70%), but their combination resulted in a higher detection rate of reclassified patients (78% vs. 67% vs. 56%).^22^ Furthermore, test results from the commercially available Progensa PCA3 test, which determines the lncRNA *PCA3* (prostate cancer associated 3) in whole urine, improved the predictive value of a clinical model consisting of age, PSAD and risk strata (AUC 0.740 vs. 0.700).^23^ In addition, Perlis et al. observed that none of the patients with a normal PCA3 score and maxPI‐RADS ≤3 were diagnosed with an upgraded PCa on repeat biopsy.^24^ In the present study, however, DLX1, HOXC6 and PCA3 from urinary cells showed no differential expression between stable and reclassified patients and thus no predictive potential. This might be explained by methodical differences, such as cohort composition, reclassification criteria, analysed urinary components (whole urine vs. cells) and qPCR conditions (commercial tests vs. non‐diagnostic TaqMan assays). Nonetheless, our previous study in urinary EVs^15^ and the present data underline the monitoring potential of molecular biomarkers for AS patients, particularly in combination with established clinical parameters. This could also lead to reduced healthcare costs per patient as already shown for the combination of SelectMDx and MRI data for PCa diagnosis.^25^ In addition, other biomarker models such as the Prostate Health Index, which is a blood test combining three different PSA derivates to improve primary detection of aggressive PCa,^26^ could be implemented in combination with clinical data to better predict reclassification and thus enhance patient stratification during AS monitoring.^13^, ^27^

Several limitations should be noted. Patients were included in this study at any time point during AS, and, thus, the interval between AS enrollment and control biopsy/urine collection was highly variable (range of 0.3–12.1 years). However, this reflects the real‐world situation since our study did not represent a prospective AS study, but a prospective liquid biopsy cohort. MRIs were not reviewed centrally and, thus, the description and reporting of MRI results bear the risk of inter‐observer variability. Similarly, patient examinations by DRE before urine collection were performed by different urologists, which could affect the yield of released urinary cells. However, while these limitations introduce a degree of variability, they also reflect the complexity of everyday clinical routine and thus underscore the practical relevance and real‐world significance of the study results. Finally, the study was conducted in a modest cohort of 74 patients. Therefore, the predictive value of the urinary transcripts and the multi‐marker model need to be validated prospectively in a larger independent cohort.

## 5 CONCLUSIONS

Overall, this study underlines the monitoring potential of molecular biomarkers for PCa patients on AS, particularly in combination with established clinical parameters. Such a multimodal approach could improve the predictability of PCa reclassification during AS and thus allow a more personalized AS approach.

## AUTHOR CONTRIBUTIONS

**Angelika Borkowetz:** Project development; funding acquisition; supervision; data collection; data analysis; manuscript writing. **Sebastian Gräfe:** Data collection; data analysis; manuscript editing. **Jeremy Kwe:** Data collection; manuscript editing. **Susanne Fuessel:** Supervision; resources; manuscript editing. **Christian Thomas:** Project development; funding acquisition; supervision; resources; manuscript editing. **Kati Erdmann:** Data collection; data analysis; manuscript writing.

## CONFLICT OF INTEREST STATEMENT

The authors declare no conflicts of interest with any financial organization regarding the material discussed in the manuscript.

## Supporting information


**Figure S1** ROC curve analysis of PSA, PSAV, MALAT1, NEAT1, STAT3 and STAT5B to discriminate between stable disease and PCa risk reclassification. *P* values were calculated by ROC curve analysis. Established (PSA, PSAV) or optimal cutoff values based on the Youden index (urinary transcripts) were used to calculate specifics of predictive performance.
**Table S2** Relative expression levels of transcripts in urinary cells from stable and reclassified patients at control biopsy and respective fold changes.
**Table S3** List of urinary transcripts without predictive potential to discriminate between stable disease and PCa risk reclassification.

**Table S1** Supporting Information.

## Data Availability

The datasets generated during and/or analysed during the current study are available from the corresponding author on reasonable request.
