# Supplementary material for: Combining PSA density and imaging data with transcript markers from urinary cells to improve prediction of prostate cancer reclassification in patients on active surveillance
Source: BJUI Compass. 2026 Aug 20;7(8):e70276. doi: 10.1002/bco2.70276 (PMC13494360; doi:10.1002/bco2.70276)
Supplement: Supplementary file 1 [file BCO2-7-e70276-s002.pdf]

Figure S1 ROC curve analysis of PSA, PSAV, MALAT1, NEAT1, STAT3 and STAT5B to discriminate between stable disease and PCa risk reclassification. P values were calculated by ROC curve analysis. Established (PSA, PSAV) or optimal cutoff values based on the Youden index (urinary transcripts) were used to calculate specifics of predictive performance.

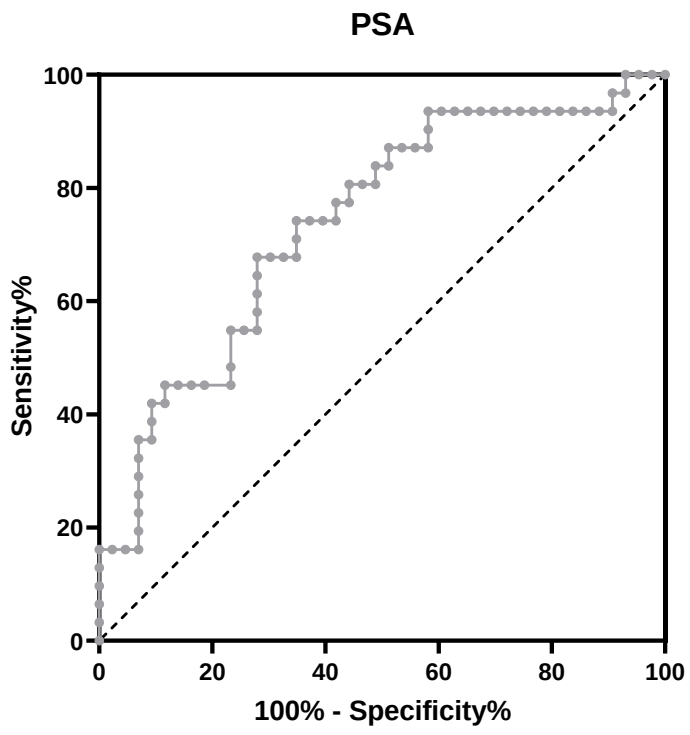

Parameter	PSA
AUC	0.739
p value	**<0.001**
Cutoff	>10 ng/ml
SNS (%)	35.5
SPC (%)	93.0
PPV (%)	78.6
NPV (%)	66.7
ACC (%)	68.9

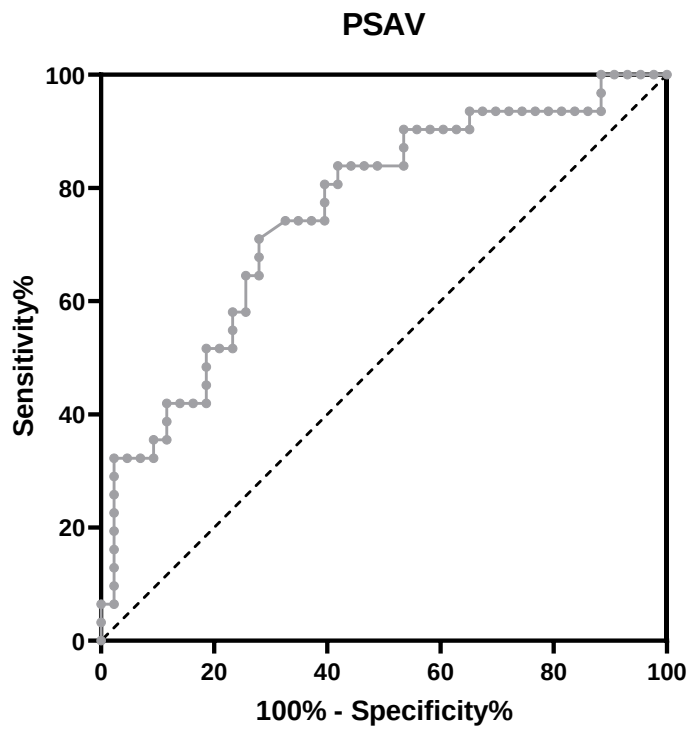

Parameter	PSAV
AUC	0.755
p value	**<0.001**
Cutoff	>0 ng/ml/year
SNS (%)	83.9
SPC (%)	53.5
PPV (%)	56.5
NPV (%)	82.1
ACC (%)	66.2

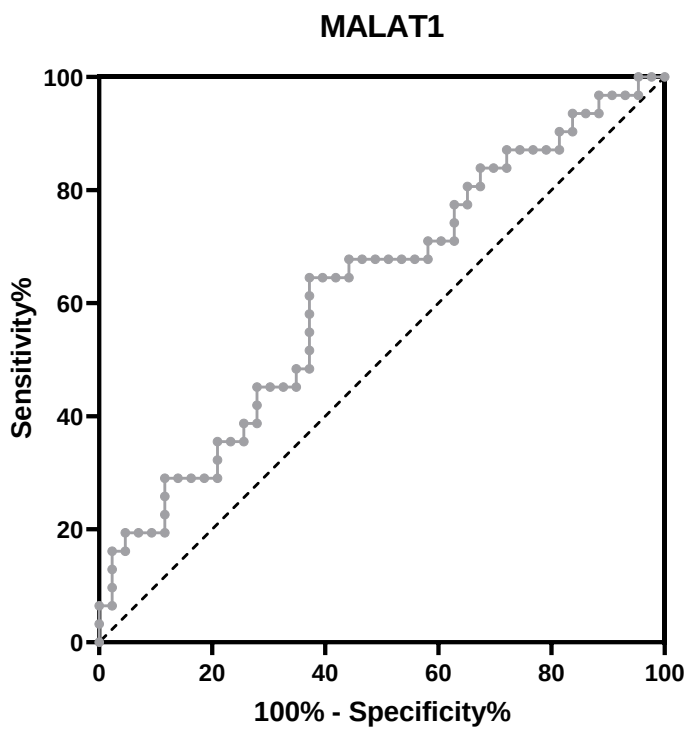

Parameter	MALAT1
AUC	0.622
p value	**0.075**
Cutoff	<111.0
SNS (%)	64.5
SPC (%)	62.8
PPV (%)	55.6
NPV (%)	71.1
ACC (%)	63.5

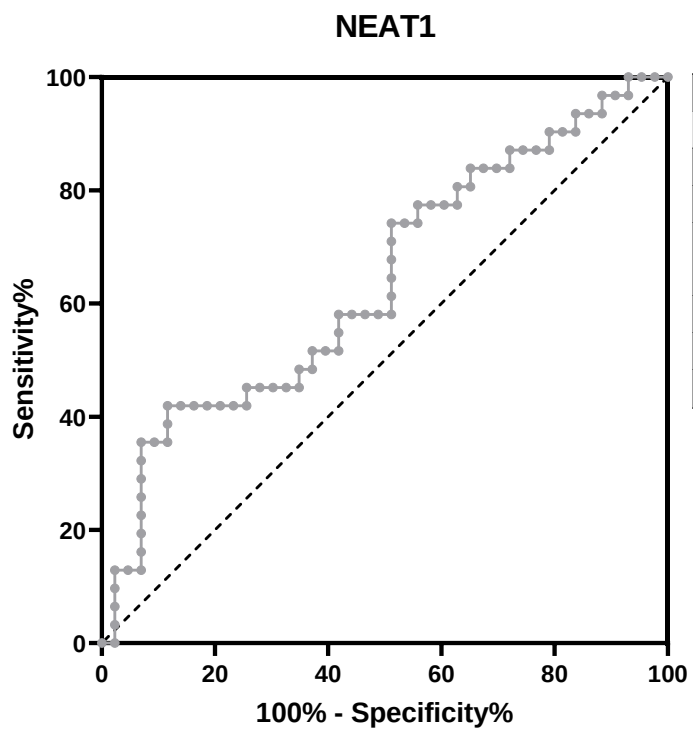

Parameter	NEAT1
AUC	0.639
p value	**0.042**
Cutoff	<25.70
SNS (%)	41.9
SPC (%)	88.4
PPV (%)	72.2
NPV (%)	67.9
ACC (%)	68.9

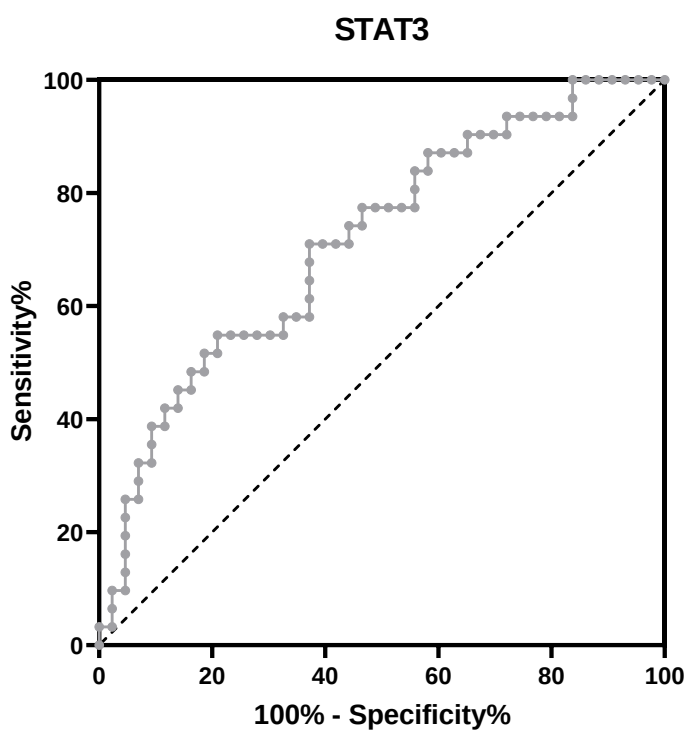

Parameter	STAT3
AUC	0.713
p value	**0.002**
Cutoff	<0.82
SNS (%)	54.8
SPC (%)	79.1
PPV (%)	65.4
NPV (%)	70.8
ACC (%)	68.9

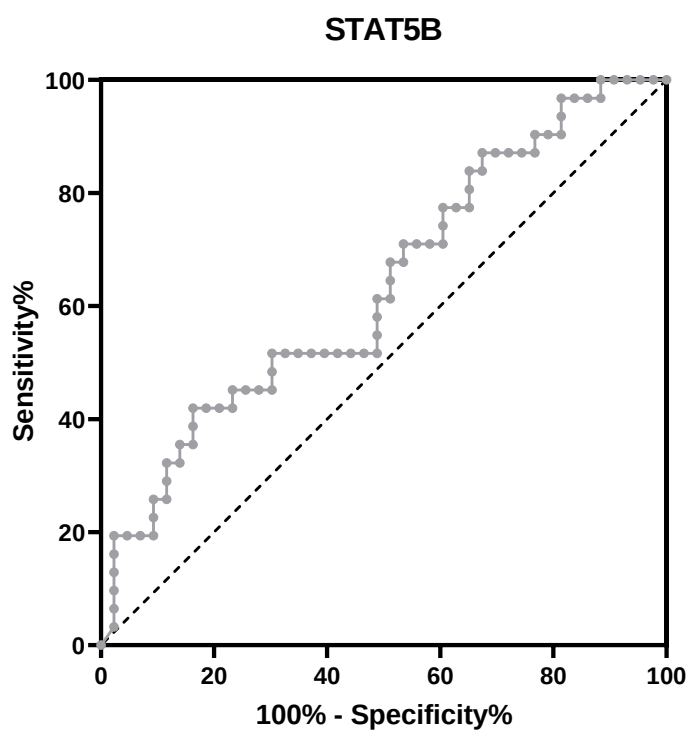

Parameter	STAT5B
AUC	0.634
p value	**0.050**
Cutoff	<0.10
SNS (%)	41.9
SPC (%)	83.7
PPV (%)	65.0
NPV (%)	66.7
ACC (%)	66.2

Table S2 Relative expression levels of transcripts in urinary cells from stable and reclassified patients at control biopsy and respective fold changes.

**Transcript**	**Median relative expression levels [a]**		**Fold change (reclassified vs stable)**	**p value [c]**
	**Stable n = 43**	**Reclassified n = 31**		
AMACR	0.0609	0.0613	1.0	0.310
BCL2	0.0569	0.0499	0.9	0.484
BCL2L1 = BCL-XL	1.1520	1.0070	0.9	0.103
CCND1	0.2376	0.3017	1.3	0.679
DLX1 [b]	0.0000 (mean: 0.0001)	0.0000 (mean: 0.0001)	NE (1.0)	0.181
ERG [b]	0.0000 (mean: 0.0034)	0.0002 (mean: 0.0023)	NE (0.7)	0.458
EZH2	0.0968	0.0911	0.9	0.580
HOXC6	0.0007	0.0003	0.4	0.528
HPN	0.0193	0.0249	1.3	0.479
KLK2	0.0031	0.0034	1.1	0.851
KLK3 = PSA	0.0069	0.0138	2.0	0.250
MALAT1	124.8000	100.2000	0.8	0.076
MCL1	5.7550	3.6940	0.6	**0.013**
NEAT1	49.0300	40.2700	0.8	**0.042**
PCA3	0.0186	0.0236	1.3	0.554
PCAT1	0.0839	0.0762	0.9	0.614
PCAT14 [b]	0.0000 (mean: 0.0008)	0.0000 (mean: 0.0052)	NE (6.3)	0.789
PCAT29 [b]	0.0006 (mean: 0.0046)	0.0000 (mean: 0.0011)	0.0 (0.2)	0.165
PCGEM1 [b]	0.0003 (mean: 0.0012)	0.0000 (mean: 0.0029)	0.0 (2.4)	0.592
Prostein = SLC45A3	0.0258	0.0333	1.3	0.400
PSGR = OR51E2	0.0005	0.0005	1.0	0.867
PSMA = FOLH1	0.0011	0.0011	1.0	0.939
SChLAP1	0.0017	0.0014	0.8	0.924
SPDEF	0.0545	0.0325	0.6	0.274
STAT3	1.4170	0.7874	0.6	**0.002**
STAT5A	0.0621	0.0492	0.8	0.139
STAT5B	0.1714	0.1216	0.7	0.050
TMPRSS2	2.1210	1.9680	0.9	0.270
TRPM8	0.0037	0.0037	1.0	0.699

NE: not evaluable.

[a] Depicted are the median relative transcript levels (normalized to geoM of PPIA, RPLP0 and TBP) in patients with stable disease and risk reclassification as well as the fold change in the reclassified group compared to the stable group ([b] mean values were also used to calculate the fold change).

[c] P values were calculated by the Mann-Whitney U test and highlighted in bold to indicate a statistical significance.

Table S3 List of urinary transcripts without predictive potential to discriminate between stable disease and PCa risk reclassification.

Transcript	AUC	95% CI	p value [a]
AMACR	0.570	0.437 to 0.704	0.306
BCL2	0.548	0.413 to 0.684	0.480
BCL2L1 = BCL-XL	0.612	0.485 to 0.740	0.101
CCND1	0.529	0.390 to 0.668	0.673
DLX1	0.570	0.439 to 0.702	0.306
ERG	0.548	0.414 to 0.681	0.487
EZH2	0.538	0.404 to 0.673	0.576
HOXC6	0.543	0.404 to 0.682	0.529
HPN	0.549	0.412 to 0.686	0.473
KLK2	0.513	0.376 to 0.650	0.848
KLK3 = PSA	0.579	0.441 to 0.717	0.250
PCA3	0.541	0.406 to 0.676	0.550
PCAT1	0.535	0.397 to 0.673	0.610
PCAT14	0.517	0.380 to 0.653	0.805
PCAT29	0.590	0.461 to 0.720	0.187
PCGEM1	0.535	0.398 to 0.672	0.607
Prostein = SLC45A3	0.558	0.423 to 0.694	0.396
PSGR = OR51E2	0.512	0.371 to 0.652	0.865
PSMA = FOLH1	0.506	0.364 to 0.647	0.935
SChLAP1	0.507	0.369 to 0.645	0.922
SPDEF	0.575	0.435 to 0.716	0.271
STAT5A	0.602	0.469 to 0.734	0.138
TMPRSS2	0.576	0.445 to 0.707	0.266
TRPM8	0.527	0.388 to 0.666	0.697

[a] P values were calculated by ROC curve analysis.
